# Pyric‐carnivory: Raptor use of prescribed fires

**DOI:** 10.1002/ece3.3401

**Published:** 2017-09-29

**Authors:** Torre J. Hovick, Devan A. McGranahan, R. Dwayne Elmore, John R. Weir, Samuel D. Fuhlendorf

**Affiliations:** ^1^ School of Natural Resource Sciences—Range Program North Dakota State University Fargo ND USA; ^2^ Department of Natural Resource Ecology and Management Oklahoma State University Stillwater OK USA

**Keywords:** *Buteo swainsoni*, disturbance, fire–grazing interaction, grassland, pyro‐diversity, tallgrass prairie

## Abstract

Fire is a process that shaped and maintained most terrestrial ecosystems worldwide. Changes in land use and patterns of human settlement have altered fire regimes and led to fire suppression resulting in numerous undesirable consequences spanning individual species and entire ecosystems. Many obvious and direct consequences of fire suppression have been well studied, but several, albeit less obvious, costs of alteration to fire regimes on wildlife are unknown. One such phenomenon is the response of carnivores to fire events—something we refer to as pyric‐carnivory. To investigate the prevalence of pyric‐carnivory in raptors, we monitored 25 prescribed fires occurring during two different seasons and across two different locations in tallgrass prairie of the central United States. We used paired point counts occurring before and during prescribed fires to quantify the use of fires by raptors. We found a strong attraction to fires with average maximum abundance nearly seven times greater during fires than prior to ignitions (before: $\bar{x}$ = 2.90, *SE* = 0.42; during: $\bar{x}$ = 20.20; *SE* = 3.29) and an average difference between fire events and immediately before fires of 15.2 (±2.69) raptors. This result was driven by Swainson's hawks (*Buteo swainsoni*), which were the most abundant (*n* = 346) of the nine species we observed using fires. Our results illustrate the importance of fire as integral disturbance process that effects wildlife behavior through multiple mechanisms that are often overshadowed by the predominant view of fire as a tool used for vegetation management.

## 1 INTRODUCTION

Fire is an ecological process that is necessary for the conservation of grassland biodiversity. Because fire has been occurring in grassland ecosystems for hundreds of millions of years, it has helped to shape global biomes and to maintain the structure and function of fire‐prone communities (Bond & Keeley, 2005; Bowman et al., 2009). As a multiscale process, fire plays a key role in the dynamics of these systems and the species that occupy them (Scholes & Archer, 1997). As a result, fire is an important component of managing fire‐dependent ecosystems (Bury, 2004; Cleary, Priadjati, Suryokusumo, & Menken, 2006; Driscoll et al., 2010). Moreover, due to decades of fire suppression and exclusion, restoration of fire is increasingly recognized as an important factor in biodiversity conservation and natural resource management (Driscoll et al., 2010). This recognition highlights the need for increased knowledge of the importance of fire regimes for wildlife conservation and is why there has been a recent increase in research focused on wildlife selection and survival in landscapes with restored fire regimes (Fuhlendorf et al., 2006; Hovick et al., 2012; McNew, Gregory, & Sandercock, 2013). Most of this research has emphasized first‐ and second‐order fire effects on organisms residing in, or dependent upon, previously burned portions of the landscape. But, research investigating the response of organisms to the actual fire event is still lacking. As a consequence, research is needed that focuses on wildlife responses to fire events (i.e., during the burning process) to help improve the conservation value of prescribed fire and to further understand the role of this important evolutionary process in wildlife behavior.

No replicated studies have examined the immediate response of organisms to fire events and many knowledge gaps exist with regard to how wildlife responds to the burning process. In particular, limited observations suggest that predatory raptors and insectivorous passerines may utilize fire events as a source for gathering necessary protein during migration or breeding (Barnard, 1987; Dean, 1987; Komarek, 1969; Stoddard, 1963). We describe this phenomenon as *pyric‐carnivory*—the response of carnivorous predators to fire in order to capture prey. While there are limited observations of pyric‐carnivory in birds, this phenomenon has never been formally named and is more commonly described postfire rather than during the burning event (Barnard, 1987; Dean, 1987; Komarek, 1969; Stoddard, 1963). As described by Dean (1987), fire can act as a beater—a source of disturbance that effectively drives organisms out of the vegetation for predators to consume. For example, kestrels and buzzards move into recently burned areas to hover for prey immediately following fire (Barnard, 1987). This occurs as vegetation is consumed by fire, exposing or injuring small mammals and insects and leaving them susceptible to predation (Conner, Castleberry, & Derrick, 2011; Letnic, Tamayo, & Dickman, 2005; Morris, Hostetler, Conner, & Oli, 2011; Morris, Hostetler, Oli, & Conner, 2011). These immediate changes to vegetation structure from fire are often prolonged when fire is coupled with large herbivore grazing, and this combined disturbance is known to alter small mammal and insect communities which make up the prey base for many grassland predators (Engle, Fuhlendorf, Roper, & Leslie, 2008; Fuhlendorf, Townsend, Elmore, & Engle, 2010; Kral, Limb, Harmon, & Hovick, 2017; Ricketts & Sandercock, 2016). Additionally, these changes in vegetation resulting from fire can influence not only resident and breeding species' interactions, but may have impacts on organisms that use burned areas for over‐wintering or in transit during migration events (Hovick, Carroll, Elmore, Davis, & Fuhlendorf, 2017).

In terms of managing fire specifically and wildlife conservation more broadly, migration is one of the least studied and understood components in the avian life cycle (Faaborg et al., 2010). Effective conservation planning needs to consider the influence of fire on nonbreeding and transient species in addition to resident and breeding species. Throughout grassland regions of the world, transient species have seasonally dependent migration events that require their short‐term dependence on systems in which they do not breed. In many circumstances, these systems are important for nutrient uptake, body maintenance, and reproductive success and survival (Morrison, Ross, & Niles, 2004; Skagen, 2006). Understanding the responses of nonbreeding, transient species to fire (and the burn event) is an additional knowledge gap where information is needed for informed and effective conservation strategies (Hovick et al., 2017; Keith, Williams, & Woinarksi, 2002).

Fire may be an instrumental process in the life history of transient species that have evolved using grassland ecosystems that have fire disturbances on some portion of the landscape each year. In particular, carnivorous raptors (i.e., birds of prey) that use grasslands systems may be highly dependent on fires to provide protein sources along migratory routes prior to breeding. Additionally, raptors are well suited for examination to the response of fire events because they represent an opportunity to make inferences about the much broader context of fire as an evolutionary process in grasslands, are easily observable and identifiable, and can provide insights into less obvious and indirect costs of the alteration to fire regimes on wildlife. Therefore, we examined raptor responses to spring and late summer fires (migration periods) in the southern Great Plains of the United States. We hypothesized that fires would attract migrating raptors, and to assess this, we surveyed raptors immediately prior to and during prescribed fires.

## 2 METHODS

### 2.1 Study area

This study was conducted at The Nature Conservancy Tallgrass Prairie Preserve (hereafter, preserve) in Osage County Oklahoma and the Oklahoma State University Cross Timbers Experimental Range (CTER) located in Payne County Oklahoma, USA. These sites are within the Great Plains ecoregion and have tallgrass prairie vegetation with small patches of cross timbers vegetation (*Quercus* spp.). The sites have diverse assemblages of tallgrass prairie plants (i.e., >200 spp.), and the most abundant species include *Andropogon gerardii*,* Schizachyrium scoparium*,* Panicum virgatum*, and *Soghastrum nutans* (Hamilton, 2007). Both sites are managed with interacting fire and grazing, with approximately one‐third of the landscape burned annually. This management framework has been in place at the preserve since the early 90s and at CTER since the late 90s. The majority (i.e., 80%) of prescribed fires are conducted during the dormant season (November‐March), with the remainder of prescribed fires being conducted during the growing season, typically in late July to early September. Both sites are grazed by domestic cattle (*Bos taurus*), and the preserve has a native bison (*Bison bison*) herd. The climate at both sites is continental, with an average frost‐free growing period of 204 days extending from April to October (Fuhlendorf and Engle 2004). Average annual precipitation for the region is 830 mm with 65% occurring during the growing season. The mean annual temperature is 15°C with the hottest temperatures occurring in August and the coolest temps in January.

### 2.2 Data collection

We quantified raptor use of 25 prescribed fires in 2013 and 2014 (*n* = 13 in 2013; *n* = 12 in 2014) using 10‐min point counts before and during prescribed fires. We placed two point count locations on the day of each fire to maximize observers' abilities to detect raptors. Points were placed >200 m apart in elevated locations along the edge of the burn area and were on the upwind or flank of the burned area to reduce limitations in visibility due to smoke. During surveys, two observers systematically scanned the burn unit and counted all perched and flying raptors, identifying them to species. Point counts were not restricted to a specific distance but only individuals flying or perching within the burn unit were counted. Through repeated scans, the observers would determine a maximum count for each species during the allotted time by taking the highest overall count for each species across the two observers and locations. This method likely produced a conservative maximum estimate but reduced our chances of biasing counts high by double counting individuals. Raptors that were unable to be identified to species were recorded as “unknown.” Point count locations were identical for pre‐ and during‐fire events and all prefire point counts were conducted within one hour before ignition of the fire. The “during” fire counts were conducted after all sides of the burn unit were fired, and the head fire was ignited and moving with the wind to ensure smoke cues had been visible to raptors and the smoke plume did not obstruct observation.

### 2.3 Data analysis

To accommodate nonindependence among before‐ and during‐fire observations, data were analyzed and reported as the difference in count per species per fire. To determine whether observed differences in counts differed from zero, we calculated 95% confidence intervals of 1000 random draws from negative binomial distribution fitted using the mean and dispersion parameter for each of the species with sufficient observations. We fit distributions using the “rnbinom” function in the R statistical environment (R Core Team, 2016).

## 3 RESULTS

**Figure 1:**
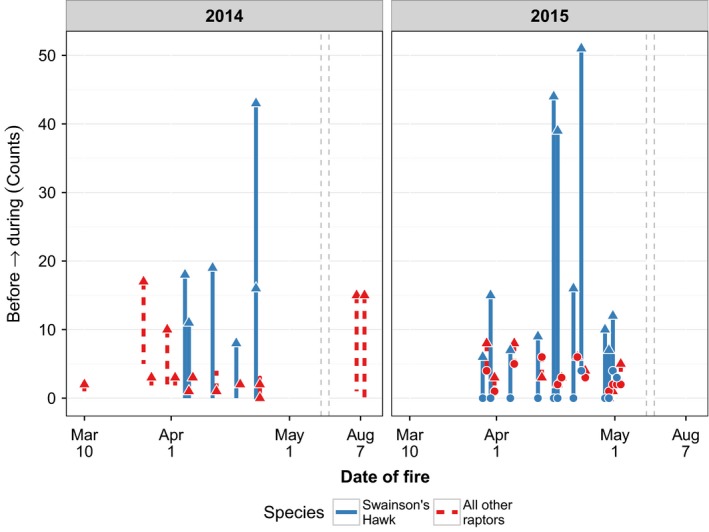
Before‐fire (dot) and during‐fire (triangle) counts for Swainson's hawk (*Buteo swainsoni*) and all other raptors observed in tallgrass prairie, Oklahoma, 2013–2014. The *x*‐axis represents the date of the fire and has a break represented as dashed lines to include summer fires conducted in 2014 that show raptor totals on the right side of the line

We detected 528 raptors made up of nine species over the course of 25 fires. Immediately prior to fires, we detected a total of 74 individuals while during‐fire events we detected 454 individuals (Table 1). We observed all species at least once during fires, two of the nine species were observed only during fires, and seven species were observed both prior to and during fires. During all 25 fires, there were more raptors detected during the fire than before ignition and this response was strongest during late April (Figure 1).

**Table 1:** Species' detections immediately preceding and during 25 prescribed fires in Oklahoma, USA (2013–2014)

Species (scientific name)	Prefire	During fire	Total
Swainson's hawk (*Buteo swainsoni*)	12	334	346
Red‐tailed hawk (*Buteo jamaicensis*)	17	39	56
Red‐shouldered hawk (*Buteo lineatus*)	2	2	4
Broad‐winged hawk (*Buteo platypterus*)	2	1	3
Rough‐legged hawk (*Buteo lagopus*)	0	1	1
Northern harrier (*Circus cyaneus*)	16	7	23
Sharp‐shinned hawk (*Accipiter striatus*)	0	1	1
American kestrel (*Falco sparverius*)	7	2	9
Mississippi kite (*Ictinia mississippiensis*)	1	30	31
Unidentified raptor	17	37	54
Total	74	454	528

**Figure 2:**
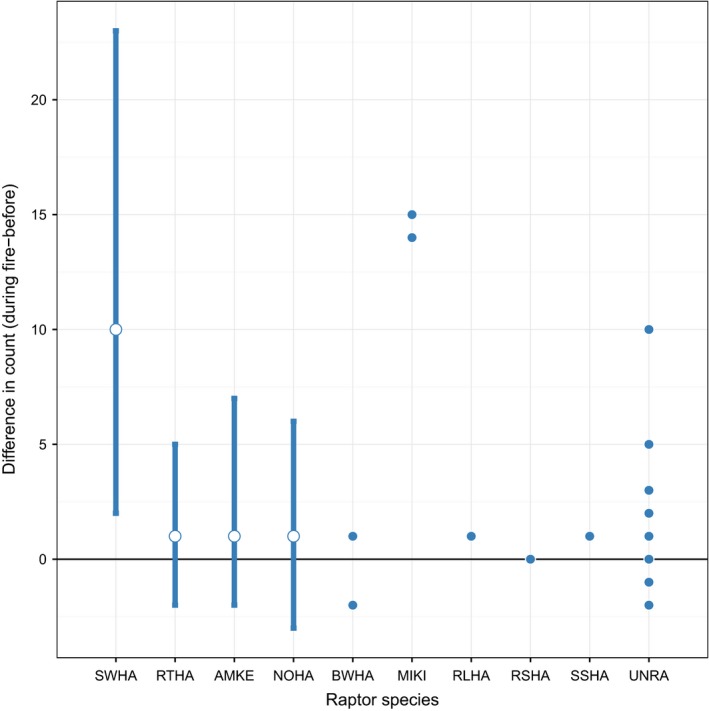
Difference between during‐fire and before‐fire counts for nine raptor species observed in tallgrass prairie, Oklahoma, 2013–2014. For five species (open circles and solid bars), simulated 95% confidence intervals indicate whether the observed value differs from zero based on negative binomial distributions fit to each species. Remaining species (solid circles) had insufficient observations to fit distributions; thus, raw observed differences are plotted. Positive values indicate attraction to fire, and negative values suggest avoidance of fire. Abbreviations: SWHA, Swainson's hawk; RTHA, red‐tailed hawk; AMKE, American kestrel; NOHA, Northern harrier; BWHA, broad‐winged hawk; MIKI, Mississippi kite; RLHA, rough‐legged hawk; RSHA, red‐shouldered hawk; SSHA, sharp‐shinned hawk, and UNRA, unknown raptor. See Table 1 for species' scientific names

We found a strong attraction to fires with average maximum abundance nearly seven times greater during fires than prior to ignitions (before: $\bar{x}$ = 2.90, *SE* = 0.42; during: $\bar{x}$ = 20.20; *SE* = 3.29) and an average difference between fire events and immediately before fires of 15.2 (±2.69) raptors. Most species—with the exception of northern harrier (*Circus cyaneus*)—showed an increasing trend toward more frequent detections during fires than prior to ignitions, but only Swainson's Hawk (*Buteo swansoni, n* = 346) showed a significant increase in during‐fire compared to before‐fire counts (Figure 2).

## 4 DISCUSSION

Fire is an important ecological process that shaped and maintained most terrestrial ecosystems worldwide (Bond & Keeley, 2005). However, changes in land use and human settlement patterns have changed fire regimes which could have unforeseen consequences on wildlife that evolved with this disturbance process. We examined how predatory raptors responded to prescribed fires in the Great Plains, USA, and found that migrating Swainson's hawks were attracted to prescribed fires (i.e., the actual burning event) and several other raptor species were detected more frequently during fires than immediately before. Our research is one of the first to quantify behavioral responses of carnivores to prescribed fires and is a phenomenon we describe as pyric‐carnivory. This research demonstrates the complexity of feedbacks between restored fire regimes and biodiversity and emphasizes how fire can positively and negatively influence food webs across all trophic levels (Bowman et al., 2016). Additionally, viewing fire as a process that varies spatially on the landscape each year but is constant temporally (i.e., occurs each year) could also be important to conservation actions for migrating species like Swainson's hawks by increasing their ability to use certain landscapes annually (Parr & Chown, 2003). Finally, this research demonstrates the importance of considering all facets of the fire process including the response of wildlife to the burning event, which is one of the most understudied components of fire ecology.

Fire is an important process in grassland and savanna ecosystems that is essential to the conservation of biodiversity (Fuhlendorf, Engle, Elmore, Limb, & Bidwell, 2012; Knapp et al., 1999; Tainton & de Mentis, 1984). Most research examining the influence of fire on birds takes place during the growing season and is generally focused on breeding species. While there is still much information to be gained on the effects of fire on breeding birds, even less is known about transient species responses to fire and in particular the response of species during the fire event. Most commonly, fire effects are focused on vegetation responses in months or years postfire (Limb, Fuhlendorf, Engle, & Miller, 2016; Pyne, 1997), but we speculate that there are many interactions that take place during the event that are important to native wildlife that have gone uninvestigated. As a multiscale process, fire plays a key role in determining the structure, functioning, and dynamics in all fire‐prone ecosystems (Scholes & Archer, 1997). Our results demonstrate one response of wildlife to fire events that has previously been observed (Barnard, 1987; Dean, 1987; Komarek, 1969; Stoddard, 1963), but never formally investigated across multiple fire events with rigorous methods to quantify the attractant effect.

**Figure 3:**
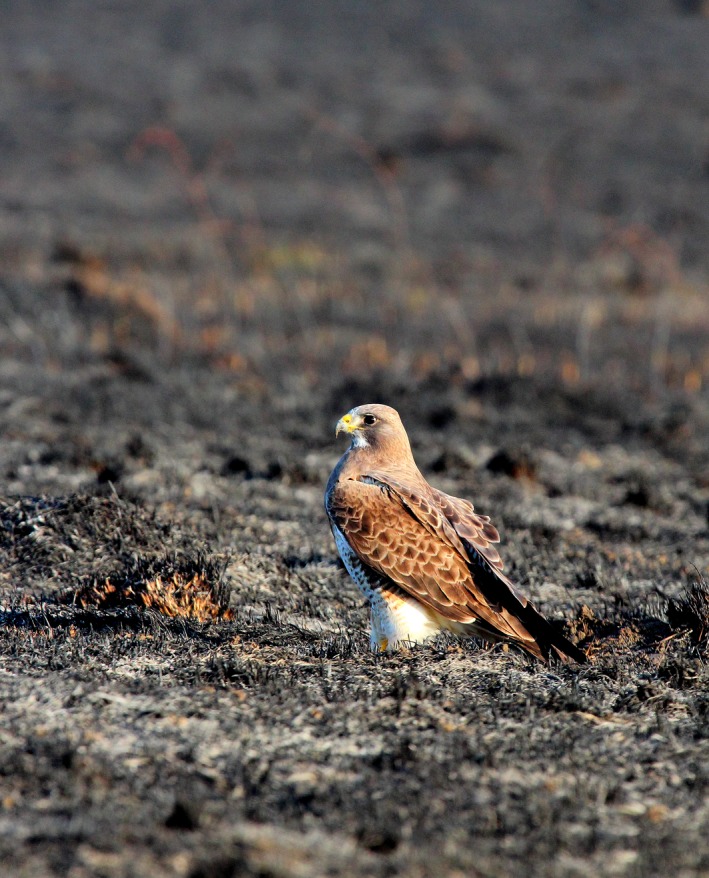
Swainson's hawk (Buteo swansoni) foraging in a recently burned area at the Tallgrass Prairie Preserve, Pawhuska, OK, USA. Photo by Torre Hovick

There was a clear increase in Swainson's hawk abundance during fires and other species also showed trends suggesting attraction to fire. The mechanism by which raptors detect fires across the landscape is unclear, but we speculate that visually oriented predators used cues from the smoke plume to exploit resources resulting from the fire. Swainson's hawks in particular are known to be daytime migrators that cover an average of 150 km/d during their northward migration from South America (Fuller, Seegar, & Shueck, 1998). Covering that amount of area coupled with the fact that they are opportunistic feeders with a large diet breadth (Bechard, Houston, Sarasola, & England, 2010) makes it likely that they have evolved a behavioral response to smoke plumes that allows them to exploit the foraging opportunities that fires provide. During our study, raptors were frequently observed consuming exposed, injured, or dead reptiles and small mammals (Figure 3). Additionally, summer fires created clouds of insects that resulted in foraging opportunities for postbreeding, migrating Mississippi kites (*Ictinia mississippiensis*; Video S1).

Our findings add to a growing body of literature emphasizing the need for restored fire regimes in grasslands, particularly the Great Plains of the central United States. Conservation concerns in grasslands commonly focus on how alteration to disturbance regimes may be contributing to declines in breeding grassland birds (Augustine & Derner, 2013; Reinking, 2005), but there has been little consideration for how these alterations could affect migrating birds that rely on grasslands to reach breeding grounds and successfully reproduce. From a land manager perspective, it is important to understand that disturbances can affect wildlife throughout their annual cycle. Specifically, fire can influence the avian community in the breeding season (Davis, Churchwell, Fuhlendorf, Engle, & Hovick, 2016; Fuhlendorf et al., 2006; Hovick, Elmore, Fuhlendorf, Engle, & Hamilton, 2015), over‐winter during the nonbreeding season (Hovick, Elmore, & Fuhlendorf, 2014), and during migration (Hovick et al., 2017). Despite being the most understudied period in the life cycle of many organisms, migration is risky and can greatly reduce survival for a population (Carlisle et al., 2009; Sillett & Holmes, 2002; Skagen & Knopf, 1993). To that end, successful migratory strategies require considerable spatial and temporal precision to reduce individual energetics and/or increase survival and reproduction (Berthold, 2001), which emphasizes the conservation value in understanding how fire regimes within migration corridors effect species.

## CONFLICT OF INTEREST

None declared.

## AUTHOR CONTRIBUTIONS

TH contributed to idea formation, data collection, interpretation of results, and writing. DM contributed to data analyses, interpretation of results, and writing. DE contributed to grant acquisition, idea formation, interpretation of results, and writing. JW contributed to idea formation, data collection, and writing. SF contributed to grant acquisition, idea formation, interpretation of results, and writing.
